# Generation of anti-Notch antibodies and their application in blocking Notch signalling in neural stem cells

**DOI:** 10.1016/j.ymeth.2012.07.008

**Published:** 2012-09

**Authors:** Ronny Falk, Anna Falk, Michael R. Dyson, Anna N. Melidoni, Kothai Parthiban, Joyce L. Young, Wendy Roake, John McCafferty

**Affiliations:** a University of Cambridge, Department of Biochemistry, Tennis Court Road, CB2 1QW Cambridge, UK; b University of Cambridge, Wellcome Trust CSCR, Tennis Court Road, CB2 1QW Cambridge, UK; c Wellcome Trust Sanger Institute, Genome Campus, Hinxton, CB10 1HH Cambridgeshire, UK

**Keywords:** Notch, Antagonistic antibody, Antibody phage-display, Neural stem cell

## Abstract

Notch signalling occurs via direct cell–cell interactions and plays an important role in linking the fates of neighbouring cells. There are four different mammalian Notch receptors that can be activated by five cell surface ligands. The ability to inhibit specific Notch receptors would help identify the roles of individual family members and potentially provide a means to study and control cell differentiation. Anti-Notch antibodies in the form of single chain Fvs were generated from an antibody phage display library by selection on either the ligand binding domain or the negative regulatory region (NRR) of Notch1 and Notch2. Six antibodies targeting the NRR of Notch1 and four antibodies recognising the NRR of Notch2 were found to prevent receptor activation in cell-based luciferase reporter assays. These antibodies were potent, highly specific inhibitors of individual Notch receptors and interfered with endogenous signalling in stem cell systems of both human and mouse origin. Antibody-mediated inhibition of Notch efficiently down-regulated transcription of the immediate Notch target gene hairy and enhancer of split 5 (Hes5) in both mouse and human neural stem cells and revealed a redundant regulation of Hes5 in these cells as complete down-regulation was seen only after simultaneous blocking of Notch1 and Notch2. In addition, these antibodies promoted differentiation of neural stem cells towards a neuronal fate. In contrast to the widely used small molecule γ-secretase inhibitors, which block all 4 Notch receptors (and a multitude of other signalling pathways), antibodies allow blockade of individual Notch family members in a highly specific way. Specific inhibition will allow examination of the effect of individual Notch receptors in complex differentiation schemes regulated by the co-ordinated action of multiple signalling pathways.

## Highlights

► Potent antibodies that antagonise mouse and human Notch signalling are generated. ► Receptor specific inhibition of Notch1 and 2 signalling is demonstrated. ► Antibody mediated inhibition of Notch influences neural stem cell differentiation.

## 1 Introduction

Notch signalling involves a bi-molecular interaction between receptor and ligand on opposing cells and influence many aspects of cell specification in the developing and adult organism. Multiple cell fate decisions are influenced by Notch including, differentiation, proliferation, apoptosis, migration and angiogenesis. Having such a central role in cell fate decisions it is unsurprising that abnormal function/expression of Notch is related to several common diseases including cancers. Notch has been shown to have both tumour suppressive and tumourigenic function in different contexts [1] and the level of expression has also been suggested to influence the degree of malignancy [2]. A range of activating mutations in the juxta-membrane and intra-cellular domains of Notch1 are known to drive the onset of T-cell acute lymphoblastic leukaemia (T-ALL) in over 55% of cases [3]. Mutations in different Notch receptors are strongly associated with other diseases. For example, a mutated Notch3 receptor is responsible for Cerebral Autosomal Dominant Arteriopathy with Sub-cortical Infarcts and Leukoencephalopathy (CADASIL) syndrome [4], Notch2 drives Allagile syndrome [5] and mutated Notch1 has been observed in aortic valve disease [6].

Many aspects of the role of Notch developmental fate determination are recapitulated in stem cell based systems. For example Notch signalling is active in neural stem cells and Notch activity maintains the self-renewable state of neural stem cells both in vivo and in vitro [7]. Notch receptor activation inhibits neurogenesis by repressing the activity of pro-neural genes to maintain a neural progenitor character of the stem cells. Knockout of Notch1 led to increased neuronal differentiation in the brains of mutant mice consistent with the view that Notch activity is needed for neural stem cell maintenance [8]. Deletion of Notch1 in the neural progenitor pool was also found to result in premature neuronal differentiation and depletion of the neural stem cells pool [9].

Notch signalling during stem cell differentiation has been studied using γ-secretase inhibitors (GSIs), reviewed in [10]. In mammals, there are four Notch receptors (Notch1–4) and five ligands (Jagged1, 2 and Dll1, 3 and 4). Despite the ability of promiscuous interactions between receptors and ligands they have their individual tissue and cellular patterns of expression and individual family members can have distinct functional roles during development [11,12]. GSIs have been used to study Notch signalling for more than a decade [13] yet they cannot distinguish between different Notch receptors since they are all γ-secretase dependent. The specificity of these inhibitors is further compromised by the fact that γ-secretase is a multi-substrate protease complex with over 60 different targets [14]. Indeed γ-secretase plays a key role in the processing of amyloid precursor protein and GSIs are currently in clinical trials for treatment of Alzheimer’s disease [15]. Diverse effects of GSIs on the many substrates of γ-secretase could lead to misleading results particularly when studying complex, multistep differentiation processes. Antibodies recognising the extracellular domain of Notch receptors provide the potential to block Notch signalling with the same degree of flexibility and temporal control as GSIs but with greater specificity. Specific, antibodies would permit reversible studies of Notch function in model systems without the need for genetic manipulation.

**Fig. 1:**
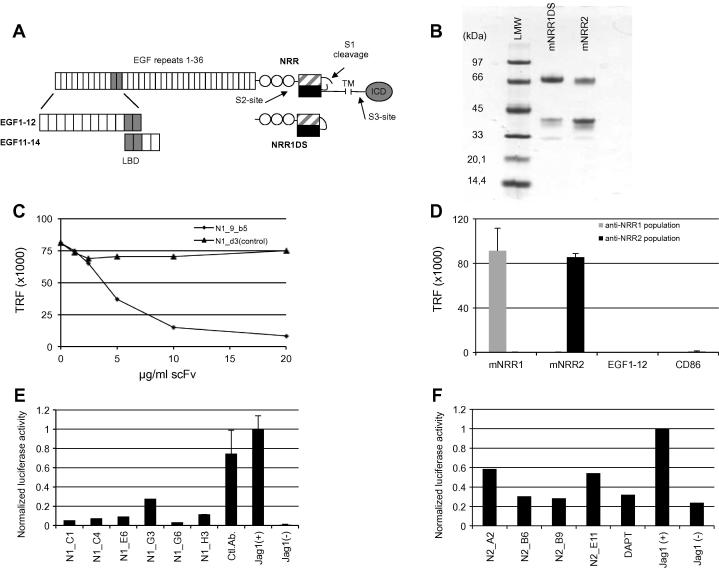
Antibodies targeting different regions of Notch are validated for target binding and functional properties. (A) Schematic outline of the Notch1 receptor with the 36 EGF-like repeats (vertical boxes) and the negative regulatory region (NRR), including the 3 LIN domains (circles) and the heterodimerisation domains (dashed and black boxes), followed by the trans membrane (TM) region and the intra-cellular domain (ICD). Ligand binding occurs around EGF domains 11–12 (grey) and induces receptor activation by exposure of the protease site, S2, in the NRR. In the full-length receptor, cleavage sites S1 and S3 are also indicated by arrows. The recombinant proteins used for antibody generation are depicted below the receptor. NRR1 was produced with or without an intact S1 cleavage site (S1). The engineered form (NRR1ΔS1) was used for selections directed to the Notch1 NRR. (B) SDS–PAGE of purified antigen proteins mNRR1ΔS1 and mNRR2. Stained protein bands in both lanes agree with theoretical size of; mNRR1DS1 (59 kDa) truncated at S1 (40 kDa), mNRR2 (57 kDa). Lower weak band at ∼30 kDa correspond to the expected size of the CD4-His10 fusion tag, occasionally observed to be cleaved off during expression of fusion proteins. (C) Identification of blocking antibodies directed to the Notch1 LBD. EGF1–12 (fused to human Fc) is incubated with Dll4 immobilised on ELISA plates. The antibody N1_9_b5 inhibits this interaction in a dose dependent way while a control antibody does not. (D) ELISA with polyclonal phage antibodies selected on mNRR1 (grey) and mNRR2 (black) showing specific binding to their respective targets. No cross reactivity between NRR1 and NRR2 populations and no binding to 2 different control proteins EGF1–12 of Notch1 and CD86 were detected. (E and F) Cell based assays for identification of blocking antibodies to Notch1 and 2. HEK293 cells expressing Notch receptors were transiently transfected with a Notch dependent Firefly luciferase reporter gene and a constitutively active Renilla luciferase gene. These were then co-cultured with cells expressing the ligand Jagged1. The ratio of luciferase activity was measured and values normalised to activity in untreated co-cultures Jag(+). Jag(−) represent the activity in unstimulated Notch-cells. (E) Six sequence unique anti-NRR1 antibodies were identified to efficiently block activation in HEK-Notch1 cells compared to an unrelated control antibody. (F) Four sequence unique anti-NRR2 antibodies that blocks activation in HEK-Notch2 cells were identified. DAPT was used as control of inhibited Notch.

Notch receptors are large, heterodimeric, transmembrane, proteins consisting of an extracellular region composed of 29–36 epidermal growth factor (EGF)-like repeats, followed by a juxta-membrane negative regulatory region (NRR) and a membrane-spanning region followed by the intra-cellular domain (ICD) that mediates signalling upon activation (Fig 1A). During maturation, the receptor is cleaved at the S1-site to form a heterodimeric, membrane spanning receptor. Ligand binding around EGF domains 11–12 of Notch causes a conformational change in the NRR domain. This exposes a proteolytic cleavage site between the extracellular and transmembrane domains (the S2-site) [16,17], which is cleaved by the matrix metalloprotease, tumor necrosis factor-α-converting enzyme (TACE). S2-cleavage generates a truncated receptor that becomes a substrate for the γ-secretase proteolytic complex, which cleaves within the transmembrane domain (S3-site). As a result the Notch ICD is released and translocates to the nucleus where it functions as a transcriptional regulator of various genes including members of the HES/HEY gene families [18].

Here we illustrate the potential of phage display to generate Notch receptor blocking antibodies, which affect signalling and linage specification of neural stem cells. The generation of antibodies targeting either the ligand binding domain (LBD) or the NRR of Notch receptors was achieved by using these regions as antigens to select single chain Fv (scFv) antibodies from a phage display library [19]. Antibodies selected on the LBD prevented receptor/ligand interaction in biochemical assays but failed to inhibit signalling in cell based assays. In contrast antibodies targeting the NRR specifically inhibited signalling through the Notch1 and Notch2 receptors and induced down-regulation of immediate Notch target gene Hes5 in both mouse and human stem cell systems. We have also shown that Notch blocking antibodies promoted neuronal differentiation of human neuroepithelial stem (NES) cells.

## 2 Material and methods

Preparative PCR was carried out with KOD Hot start polymerase (Novagen) and analytical PCR with Taq PCR master mix (Qiagen). Primer sequences are shown in Supplementary Table 1. Restriction enzymes were purchased from New England Biolabs (NEB). QiaPrep spin columns (Qiagen) were used for clean up of DNA according to manufacturer’s recommendations. Alignment of NRR proteins was performed with Vector NTI software (Invitrogen). The GSI compound DAPT (γ-Secretase Inhibitor IX, Cat #565770, Calbiochem) was used as control of Notch inhibition as indicated.

### 2.1 cDNA constructs and reporter cell lines

Human embryonic kidney (HEK) 293T cells stably expressing full-length mouse Notch1 (HEK-Notch1) and human Jagged1 (HEK-Jag1) and HEK293 cells expressing mouse Notch3 (HEK-Notch3) were kindly provided by Professor Urban Lendahl, Karolinska Institute, Sweden. Full-length mouse Notch2 cDNA (IMAGE:100069450, described at www.ncbi.nlm.nih.gov/nuccore/225000669) was cloned into the GATEWAY destination vector pPyCAG-IRES-Blast (a kind gift from Dr. Joerg Betschinger, CSCR, Cambridge, UK). This expression construct was transfected into HEK293T cells using FuGene6 transfection reagent (Roche) according to manufacturer’s recommendations. After selection in media supplemented with 7 μg/ml Blasticidin, HEK-Notch2 cells stably expressing Notch2 were isolated from a single colony.

Reporter cells were cultured in Dulbecco modified eagle medium (DMEM), high Glucose and l-Glutamine (PAA, UK) supplemented with 10% FBS (PAA), 1% Pen-Strep (PAA) and the following antibiotics for respective cell line, 0.5 μg/ml Puromycin (HEK-Notch1 and HEK-Jag1), Neomycin (HEK-Notch3) and 5 μg/ml Blasticidin (HEK-Notch2). Cells were maintained in 5% CO_2_ at 37 °C and cultured in tissue culture treated plastics.

### 2.2 Cloning of Notch domains

The Notch1 LBD encompassed by EGF domains 11–14 and mNRR1 and mNRR2 were isolated from a universal mouse cDNA library and hNRR1 and hNRR2 were isolated from the Universal II human cDNA library (Clontech). For gene amplification, a previously described nested PCR strategy was used [20]. For the first PCR (PCR1), outer primers isolated the target DNA flanked by 30–40 nucleotides at both ends. This product was then further amplified using nested primers in a second PCR. Isolated mouse NRRs, encoding amino acids D1429–P1721 (Notch1) and P1403-Q1679 (Notch2), were Gateway adapted and cloned into the vector pTT3DestrCD4(d3+4)-His10 as previously described [21]. Human gene fragments encoding amino acids E1446–Q1733 of hNRR1 and P1422–Q1677 of hNRR2, were cloned into the XhoI/NotI sites of vector pBIOCAM4 (unpublished). The vector pBIOCAM4 is based on the pCMV/cMyc/ER plasmid (Invitrogen) and introduces a C-terminal fusion tag consisting of the rat CD4 domain (3 + 4), His6 and 3xFLAG to the NRRs. The S1 cleavage sites were deleted from human and mouse NRR1 constructs by PCR using forward primers, F mNRR1dS1 and F hNRR1dS1, annealing immediately downstream and reverse primers, R mNRR1dS1 and R hNRR1dS1, immediately upstream of the S1-site. PCR products were treated with DpnI restriction enzyme to degrade remaining parental plasmid before the amplified DNA was re-circularised by T4 DNA ligase (NEB). In the resulting constructs, amino acids 1651–1654 (RQRR) in mouse NRR1 and 1662–1665 (RRRR) in human NRR1 were removed. Sequencing was used to confirm the correct inserts were cloned.

### 2.3 Mammalian protein production

A fusion protein consisting of EGF domains 1–12 of murine Notch1 fused to a human Fc domain (EGF1–12) was used for selections (R & D systems, Cat #1057-TK). The NRR and LBD domains were produced by transient expression in suspension adapted HEK293E cells as previously described [21,22].

For purification Ni–NTA purification resin (Qiagen) and culture supernatants were mixed for 1 h and transferred to Proteus 1-step midi spin columns (Generon, UK) and unbound proteins were washed out with phosphate buffered saline (PBS) supplemented with 200 mM NaCl and 20 mM imidazole, pH 8. Bound proteins were eluted in fractions with elution buffer (50 mM Tris–HCl, 400 mM imidazole, pH 8). Protein containing fractions were pooled and dialysed against PBS with GeBaflex dialysis tubes, 3.5 kDa MWCO (Generon) and analysed for purity and concentration by SDS–PAGE, Western blot and spectrophotometry. For SDS–PAGE, purified proteins were separated on a NuPage™ Novex 4–12% Bis-Tris gel (Invitrogen) at 200 V and protein bands visualised by staining in SimplyBlue™ SafeStain (Invitrogen) according to manufactures recommendations.

For verification of active preparations of LBD protein (EGF11–14), ELISA plates were coated overnight with recombinant mouse Dll4 or CD28 (R & D systems). Following blocking with PBS containing 3% Marvel® (PBSM), dilutions of EGF11–14 were added to the coated wells. Any binding of the recombinant LBD protein was detected via the CD4 tag using alkaline phosphatase labelled anti-CD4 MAb followed by TMB substrate application.

### 2.4 Antibody selection

Antibodies were selected from a phage display scFv antibody library [19,23] by 2 rounds of selection as previously described [19]. For antigens fused to CD4 (domain 3+4) His10, a de-selection step against this fusion tag was performed prior to the first selection round. In subsequent selection rounds, antigens (mouse NRRs or Notch1 EGF1–12) were coated at 5 μg/ml to separate MaxiSorp tubes (Nunc Inc.).

### 2.5 Binding specificity of scFv-phage populations in ELISA

Polyclonal phage ELISAs were done to verify target binding of selected scFv populations. ELISA plates for analysis of anti-NRR populations were coated overnight at 4 °C with 5 μg/ml NRR along with 2 unrelated control proteins, murine Notch1 (EGF1–12, R&D systems) and CD86 (previously produced as in [21]). ELISA wells were blocked with PBSM and a 1 in 10 dilution (relative to initial culture volume) of phage-scFv in PBSM was added to each well and further incubated at RT for 1 h. After consecutive washing with PBS containing 0,05% Tween 20 (PBST) and PBS, bound phage particles were probed with a mouse-anti-M13 antibody (GE Healthcare) followed by a europium labelled anti-mouse-IgG (Perkin Elmer). To detect antibody binding, 100 μl enhancer solution (Perkin Elmer) was added to the wells 10 min before measuring the time resolved florescence (TRF) in a Fusion plate reader instrument (Perkin Elmer).

### 2.6 Sub-cloning expression and screening of antibodies

For bacterial expression, selected antibody genes were isolated by PCR as previously described [19]. Amplified scFv genes were cloned into the NcoI/NotI sites of either the bacterial expression vectors pSANG10–3F (anti-NRR populations) or pSANG14–3F (anti-EGF1–12 population) [24]. The sub-cloned antibody populations were transformed into BL21 (DE3) *Escherichia coli* cells and spread on 2TY-agar plates supplemented with 2% glucose and 50 μg/ml Kanamycin. Small scale expression and ELISA screens were carried out as previously described [19,23].

For bacterial production of antibodies used in functional screens (Section 2.8), antibody clones were grown in 5 ml auto-induction medium supplemented with 50 μg/ml kanamycin in 24-well plates and periplasmic extracts prepared as previously described [24]. For antibody purification, Ni–NTA super-flow resin (Qiagen) was equilibrated in 2xPBS supplemented with 10 mM imidazole, pH 8 and 50 μl resin (bed volume) dispensed to wells of a 96-well filter plate, Whatman Unifilter, 800 μl, 25 μm, polypropylene (GE Healtcare). Periplasmic material was added to wells containing Ni–NTA resin and allowed to mix for 1 h at RT. Unbound material was pulled through the filter by gentle centrifugation followed by addition of 600 μl PBS supplemented with 20 mM imidazole, pH 8, for further washing. When all washing buffer was pulled through, the filter plate was placed on top of a Kingfisher 96 well collection plate (Thermo Scientific) and the purified antibodies were eluted in 200 μl elution buffer (PBS supplemented with 200 mM NaCl and 250 mM imidazole, pH 8) by employing gentle centrifugation. Recovered antibodies were analysed with SDS–PAGE.

Identified blocking antibodies (Section 3.2.1) were reformatted as scFv-Fc-fusions by sub-cloning into the plasmid pBIOCAM5–3F (unpublished) and the resulting mammalian expression constructs named according to respective antibody clone, e.g. pBIOCAM5-N1_E6. Along with the blocking antibodies, an anti-Notch3 antibody, N3(E10), previously selected as a scFv antibody against murine Notch3 (R&D systems) (unpublished), was also converted to scFv-Fc for use in flow cytometry (Section 2.12). Expression from pBIOCAM5–3F is under the CMV promoter and provides a C-terminal fusion partner, consisting of human Fc, His6 and 3xFLAG, to the antibody gene. Antibodies were formatted as scFv-Fc fusions in subsequent ELISA and cell based signalling assays.

To determine cross species binding of the sub cloned anti-NRR1 and anti-NRR2 antibodies, ELISA plates were coated with mouse or human NRR proteins (1 μg/ml). Serial dilutions of antibodies (0.1–5 μg/ml) were applied for 1 h at RT in PBSM and washed with PBST and PBS. Binding of the Fc-fused antibodies in ELISA were detected with a europium labelled anti-human-Fc antibody (Perkin Elmer) and monitored with TRF as described in Section 2.5.

### 2.7 Cloning of a blocking Notch3 antibody

The gene encoding the variable heavy (VH) and variable light (VL) chains of the blocking Notch3 monoclonal antibody A4 described by [25] (Patent No: WO 2008/076960 A2) was synthesised (Geneart) with flanking NcoI and NotI restriction sites (at the 5′and 3′end respectively) and a linker region between the heavy and light chains were introduced as indicated; 5′-GGTACCGCCATGGCC-**VH**-CTCGAGGGTGGCGGAGGAAGTGGAGGCGGAGGATCAGGCGGCGGAGCTAGC-**VL**-GCGGCCGCAGAGCTC-3′. The obtained antibody construct (denoted N3_mAb) was cloned into plasmid pBIOCAM5–3F for expression in HEK293E cells (see Sections 2.3 and 2.6).

### 2.8 Luciferase reporter-gene assays

Signalling in Notch expressing cells was activated either by co-culturing with HEK-Jag1 cells or by immobilised ligand. Prior to co-culturing, Notch expressing cells (Section 2.1) were co-transfected with the reporter plasmids, 12xCSL-luciferase [26] and pRL-CMV (Promega). Expression of Firefly luciferase from 12xSCL-luciferase is dependent on Notch receptor activation while Renilla luciferase is constitutively transcribed from pRL-CMV and used to normalise the assay. Notch expressing cells were seeded at 30% confluence one day before transfection. Transfections were done with Fugene6 (Roche) according to manufacturer’s recommendations. For analyses using transient expression of mouse Notch3 in HEK293T cells, the plasmid pcDNA3-Notch3 (a gift from Professor U. Lendahl, Karolinska Institute, Sweden) was co-transfected alongside the luciferase constructs. The day after transfection, cells were seeded into a 96-well culture plate and allowed to adhere for 6–8 h. DAPT or antibody preparations were added to wells before the addition of HEK-Jag1 or HEK293T cells (for non-activated controls) at a 1:1 cell ratio. The final antibody concentration was kept below 10 μg/ml in all wells. Co-culturing was continued for 14–18 h and luciferase activity was analysed with a dual-luciferase reporter assay system (Promega) according to manufacturer’s protocol using a Glomax instrument (Promega).

For activation with recombinant ligand, 96-well culture plates were coated with 50 μg/ml protein G (Zymed Laboratories) overnight at RT. Coated wells were washed with PBS, blocked with 10 mg/ml BSA in PBS for 2 h and incubated with Jagged1-Fc (R&D systems) or a human Fc fragment (CromPure human IgG fragment, Jackson ImmunoResearch) diluted to 1 μg/ml in PBS supplemented with 0.1% BSA. Antibodies were either added to coated wells or pre-mixed with transfected Notch cells (see Section 2.10.1) prior to the addition of Notch expressing cells to wells.

### 2.9 Stem cell culture

Human neuroepithelial stem (NES) cells AF22 [27] were expanded on poly-l-ornithine and 10 μg/ml laminin (Sigma) coated plates in DMEM/F12, 2 mM l-glutamine, 1.6 g/l glucose, 0.1 mg/ml penicillin/streptomycin, N2 supplement (1:100) (Invitrogen) supplemented with 10 ng/ml fibroblast growth factor-2 (FGF2), 10 ng/ml EGF (R&D systems) and 1 μl/ml B27 (Invitrogen) [27]. Confluent cultures were trypsinised and seeded at a ratio of 1:3. Media was changed every day and supplemented with Fc-fused anti-Notch antibodies as indicated: N1_E6 (1 μg/ml), N2_B9 (5 μg/ml), N3_mAb (5 μg/ml), DAPT (2 μM, 1:500), DMSO (1:500).

Mouse NS cells Cor8.2 were cultured on gelatin (0.1%, Sigma) in RHB-A media (Stem Cell Science) supplemented with 10 ng/ml FGF2, 10 ng/ml EGF (both from R&D systems) [28]. Media was supplemented with blocking antibodies at a concentration of 10 μg/ml.

### 2.10 Stem cell differentiation

Neuronal differentiation of NES cells was done as previously described [27]. This involved removing the growth factors FGF2 and EGF from the media and culturing the cells in Neurobasal media supplemented with B27 (1:50, Invitrogen) and DMEM/F12 media supplemented with N2 (1:100) mixed at a 1:1 ratio. Media was changed every second day and supplemented with Fc-fused anti-Notch antibodies as indicated:

N1_E6 (1 μg/ml), N2_B9 (5 μg/ml), N3_mAb (5 μg/ml), DAPT (2 μM, 1:500), DMSO (1:500).

### 2.11 cDNA synthesis and quantitative real-time (qRT) PCR

Proliferating or differentiating mouse NS or human NES cells were harvested in RLT buffer containing ß-mercaptoethanol (1:100) and RNA was prepared according to the manufacturer’s protocol (RNeasy, Qiagen). RNA was eluted in 50 μl water and 1 μg RNA was used to prepare cDNA using random primers and Superscript III Reverse Transcriptase (Invitrogen). For PCR amplification, cDNAs were amplified using Taqman master mix (ABI), specific primers (Sigma) and Probes (Universal Probe Library). Primers and probes are listed in supplementary Table 2. Reactions were run on a StepOne machine from Applied Biosystems and the data were analysed using Excel.

### 2.12 Flow cytometry

HEK-Notch1, HEK-Notch2 and HEK-Notch3 cells were dissociated with Hanks dissociation buffer (Gibco), washed and resuspended in DMEM media. Cells were stained for 1 h with 1 μg/ml of respective anti-Notch antibody in 50 μl DMEM. After washing with 10 ml DMEM, Notch binding antibodies were probed either with a biotinylated anti-FLAG antibody (Sigma) followed by streptavidin–phycoerythrin (Peirce) or by a phycoerythrin-conjugated anti-Fc antibody (Jackson Immunoreserch laboratories). After each incubation the cells were washed in 10 ml DMEM and finally resuspended in 500 μl DMEM for analysis. Dead cells were labelled with TO-PRO 3 (Invitrogen) immediately before analysis (Cyan, DAKO cytomation).

## 3 Results

### 3.1 Generation of antibodies recognising the ligand binding domain (LBD) of Notch1

Our initial strategy was to modulate receptor activity by creating antibodies that compete with Notch ligands for binding to the LBD. Mutational studies have shown that EGF repeats 11 and 12 of the Notch receptor represent the LBD [29]. Initially two rounds of selection were carried out on a commercially available form of Notch1, which incorporated EGF-like domains 1–12 fused to a human Fc domain (EGF1–12). An antibody phage–display library of 10 billion clones was used for antibody selection [19]. In this library antibodies are presented in the form of scFv. The resultant scFv population was sub-cloned for bacterial expression and 124 target-binding clones with unique sequence were identified in ELISA. These included 70 clones that specifically bound Notch1 without cross reactivity to the corresponding extracellular regions of Notch 2 or 3 (data not shown).

To identify antibodies specifically binding to the LBD, a shorter region of Notch1 (EGF11–14) (Fig. 1A) was cloned and protein expressed. The presence of correctly folded protein was confirmed by binding of EGF11–14 preparations to immobilised ligand (Dll4) in ELISA (data not shown). Antibodies selected on EGF1–12 were screened for binding to immobilised EGF11–14. This led to the identification of 3 clones, including N1_9_b5, that bound both targets, presumably by recognising epitopes from EGF domains 11–12 which is present on both forms. To determine if the antibodies binding EGF11–14 interfered with ligand binding, an ELISA-based binding assay was developed which recapitulated the ligand/receptor interaction of Notch1 and Notch2 (EGF1–12) with Dll4. Binding of EGF domains 1–12 of Notch3 to ligand was not observed in agreement with existing findings where a different set of EGF-like repeats has been suggested to be involved in ligand binding [30]. Addition of the antibody N1_9_b5 to the receptor binding assay prevented binding of Notch1 to Dll4 with an half maximal inhibition concentration (IC_50_) of 130nM (Fig. 1C). A control antibody, binding to Notch1 outside the LBD, did not influence binding of Notch1 to Dll4. N1_9_b5 did not inhibit the interaction of Notch2 with Dll4 in keeping with the Notch1 specificity previously seen in ELISA. The N1_9_b5 antibody was tested for its ability to block Notch signalling in a cellular reporter assay, based on expression of a Notch dependent luciferase reporter construct (described in 3.2.1 below). In this assay, the antibody failed to block Notch signalling.

### 3.2 Generation of antibodies to the negative regulatory region (NRR) of Notch1 and Notch2

Antibodies directed to the NRR of the Notch receptor (Fig. 1A) represent another route to block Notch signalling. During expression and transport of Notch receptors to the cell surface, there is constitutive cleavage of the receptor by a furin protease at a juxtamembrane proteolytic cleavage site (S1) [31]. The resulting fragments remain associated through non-covalent association of the heterodimerisation domains. This heterodimerisation domain together with three overlying LIN domains constitutes the NRR. Ligand activation causes a conformational change in the NRR domain leading to exposure of additional cleavage sites (S2 and S3) [16,17] eventually resulting in release of the ICD. The NRR of human Notch has previously been targeted by antibodies [25,32,33]. In this study antibodies were generated against the NRR of mouse Notch1 and 2.

Antigens for selection were generated by transient transfection of HEK293E cells. The NRR regions of mouse Notch1 (mNRR1) and mouse Notch2 (mNRR2) were fused at their C termini with a decahistidine tag for purification and a CD4 fusion partner previously shown to enhance expression in this system [21]. During antigen purification there is the potential for loss of the N terminal fragment of the NRR, since cleavage at the S1 site means there is a reliance on non-covalent association to retain the untagged N terminus. To circumvent this, an NRR1 construct was made where the S1 site was deleted. As a result the proportion of non-cleaved NRR increased from approximately 30% to 70% of the recovered material. Deletion of residues corresponding to the S1 site did not influence the overall protein yield as determined by SDS–PAGE (Fig. 1B) and approximately 5–10 mg protein was recovered per litre culture supernatant with 90% purity. This material was successfully used in subsequent selection and ELISA experiments.

Antibody populations targeting the NRRs of Notch1 and Notch2 were isolated from the phage display library after two rounds of selection [19,23]. Polyclonal phage ELISA confirmed that binding antibodies had been selected (Fig. 1D). The populations showed little or no binding to two control protein antigens, (EGF1–12 of mouse Notch1 and CD86), or to the alternative NRR molecule indicating that the selected populations are highly specific for their respective targets. Following sub-cloning for soluble expression in bacteria [24], a total of 470 clones from each population were expressed, tested and ranked according to signal intensity in ELISA (Supplementary Fig. 1). The 96 clones with highest signal intensity from each set were chosen for further characterisation.

#### 3.2.1 Identification of antibodies blocking Notch1 and Notch2 signalling

In our earlier efforts targeting the Notch LBD, antibodies that interfered with ligand/receptor interaction were identified in ELISA-based ligand binding assays. Targeting the NRR, however, requires direct screening in cellular assays based on measuring Notch-controlled reporter gene expression. This was done using a co-culture system where HEK293T cells expressing the ligand Jagged-1 (HEK-Jag1 cells) and HEK293T cells expressing either Notch1 (HEK-Notch1) or Notch2 (HEK-Notch2) were co-cultured. The Notch expressing cells were transiently transfected with a luciferase reporter driven by the Notch responsive CSL promoter so that ligand activation of Notch resulted in activation of luciferase expression. Blocking antibodies are expected to prevent or reduce this activity. The GSI-compound DAPT, inhibits signalling from all 4 Notch receptors and was used as a positive control.

The top 96 antibodies selected on either Notch1 or Notch2 were expressed and purified from *E. coli* and investigated for their ability to modulate Notch1 and Notch2 signalling respectively. A number of blocking antibodies were identified and sequence analysis revealed that six unique anti-Notch1 antibodies were isolated (Fig. 1E and Supplementary Fig. 6). Antibody clone N1_E6 was independently isolated six times with all others appearing once. For Notch2, four unique blockers were each isolated as single hits (Fig. 1F and Supplementary Fig. 6).

The selected antibodies were originally produced in *E. coli*. To exclude the possibility of interference from lipopolysaccharides or other remaining bacterial products in cell based experiments, the functional antibodies were also expressed in HEK293 cells. This was achieved by sub-cloning the scFv gene into a mammalian expression vector (pBIOCAM5–3F) which fuses the scFv to a C-terminal human Fc domain with a tri-FLAG tag and hexahistidine tag. This has the additional benefit of increasing the valency of the selected scFv. Following sub-cloning and transient expression the resultant bivalent antibodies were successfully produced in HEK293E cells (1–10 mg/l) and recovered at more than 90% purity after affinity purification and dialysis (data not shown). The reformatted antibodies were re-evaluated for functionality and maintenance of antagonistic activity was confirmed for all antibody clones (Supplementary Fig. 2A and B). Similar results were seen when the cellular orientation was reversed i.e., when HEK-Jag1 cells were seeded first, followed by addition of receptor expressing cells. This confirmed that blocking occurred irrespective of the cellular orientation (data not shown). In addition, receptor-specific blocking of Notch1 and 2 could be demonstrated when receptor activation was stimulated using the immobilised ligand Jagged1 rather than cellular expressed ligand. (Supplementary Fig. 3A–C).

#### 3.2.2 Specificity and potency characterization of selected antibodies

**Fig. 2:**
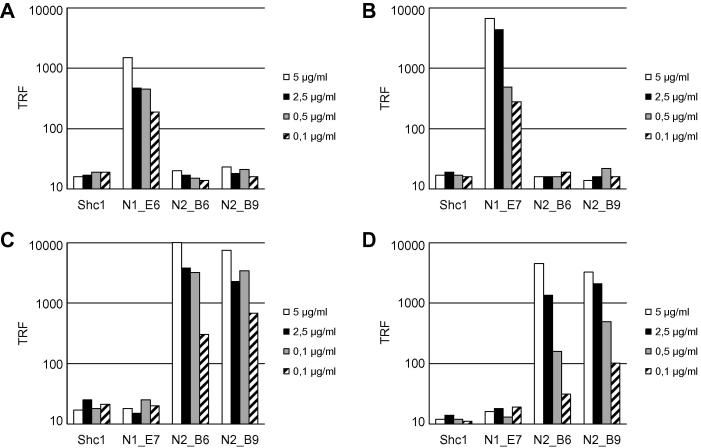
Specificity of anti-Notch1 and anti-Notch 2 blocking antibodies. ELISA experiments were carried out with serial dilutions of antibodies on immobilised NRR domains of mouse Notch1 (A), human Notch1 (B), mouse Notch2 (C) and human Notch2 (D). This confirms that the anti-Notch1 antibody N1_E6 bind to both mouse mNRR1 and human hNRR1 (A-B) while the anti-Notch2 antibodies N2_B6 and N2_B9 do not. Conversely, N2_B6 and N2_B9 but not N1_E6 bind to NRR2 of both mouse and human origin (C–D) origin. A control antibody directed to the protein Shc1 did not bind to any of the immobilised NRRs.

Amino acid alignment of human and mouse NRRs (Supplementary Fig. 4) demonstrates high identity between human and mouse orthologues of NRR1 (90%) and NRR2 (88%). In contrast identity between the paralogues of NRR1–4 within a species is more distant (e.g. 46% identity between mouse NRR1 and NRR2). ELISAs were carried out to determine if the anti-Notch1 and anti-Notch2 antibodies cross-reacted with equivalent human Notch genes and to determine if they were specific within different paralogues in the same species (Fig. 2A–D). The result confirmed cross-species reactivity of the antibodies between mouse and human Notch1 and 2. Further, the anti-Notch1 antibodies do not bind Notch2 and the Notch2 antibodies fail to bind to Notch 1. Thus the cross-species reactivity demonstrated the utility of these antibodies in cell systems of mouse and human origin.

**Fig. 3:**
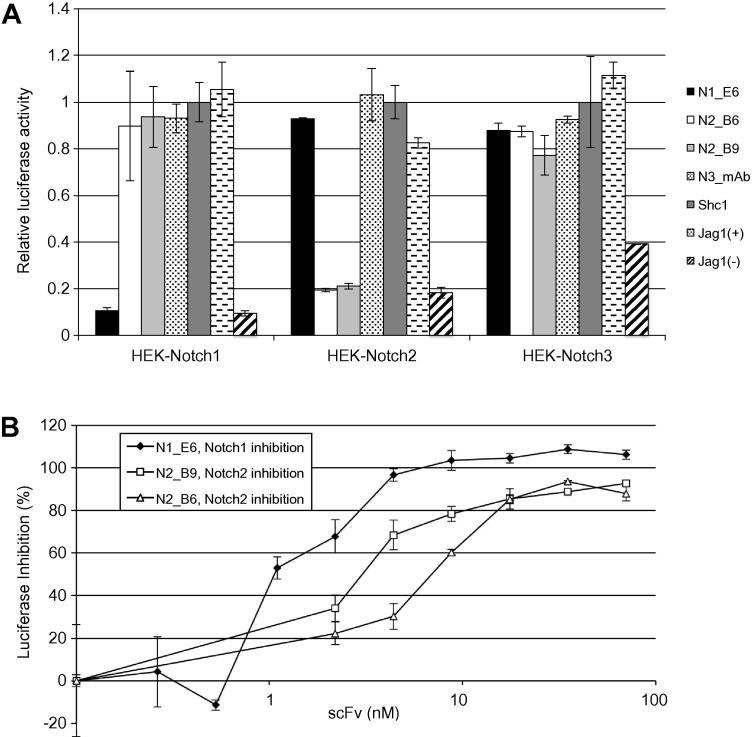
Receptor specific and dose dependent inhibition of Notch signalling between cells. Luciferase based co-culture assays were conducted with cells expressing the ligand Jagged1 and cells expressing different full-length Notch receptors (HEK-Notch1, HEK-Notch2 and HEK-Notch3). (A) Samples tested were anti-Notch1 antibody N1_E6, anti-Notch2 antibodies N2_B6 and N2_B9, the anti-human Notch3 antibody N3_mAb. Controls are non-activated, Jag1(-), and untreated, Jag1(+), cultures. The ratio of Firefly/Renilla luciferase activity is expressed relative to that of a control antibody (Shc1). (B) Dose response curves were generated by titration of N1_E6 on HEK-Notch1 and antibodies N2_B6 and N2_B9 on HEK-Notch2. The relative luciferase activity in non-activated cells were used to reflect 100% inhibition and the standard deviations were calculated to be 100 ± 6,65% for HEK-Notch1 (D) and 100 ± 2,51% for HEK-Notch2.

Specificity in signal blocking was confirmed using co-culture assays employing cells expressing Notch 1, 2 or 3 and HEK-Jag1 cells. Expression of Notch in these cells was confirmed by flow cytometry analysis (Supplementary Fig. 5A–C). The anti-NRR1 antibody, N1_E6, specifically stained cells expressing Notch1 but not Notch 2 or Notch 3 (top panel). Specific staining with the N2_B9 antibody confirms Notch2 expression in HEK-Notch2 cells (second panel). Using an anti-Notch3 antibody (N3_E10) derived by phage selection on Notch3 (unpublished) specific staining was observed only on HEK-Notch3 cells (lower panel). Thus these reagents provided a set of antibodies that enabled the confirmation of Notch1–3 expression by flow cytometry. Although the HEK-Notch3 cells appear to be a heterogeneous population from the flow cytometer data, functional Notch3 signalling could be activated by co-culturing with HEK-Jag1 cells (Fig. 3A) or using recombinant ligand (Supplementary Fig. 3C). These results are in line with previous findings utilising the same Notch3 cell line [34].

By using these cells in co-culture assays, it could be clearly demonstrated that antibody blockade of Notch signalling with anti-Notch1 or anti-Notch2 antibodies is highly specific (Fig. 3A). The anti-NRR1 antibody N1_E6 completely blocked Notch1 without interfering with Notch2 or Notch3 signalling. Similar, the anti-NRR2 antibodies N2_B6 and N2_B9 completely inhibited activation of Notch2 without affecting the activation of either Notch1 or Notch3 (Fig. 3A and Supplementary Fig. 3). The potency of blocking antibodies was determined by testing a range of antibody concentrations in the co-culturing assay. The IC_50_ of blocking antibodies were determined to be below 1.1 nM (0.12 μg/ml) for N1_E6 and 8.8 nM (1.0 μg/ml) for N2_B6 and 4.4 nM (0.5 μg/ml) for N2_B9 (Fig. 3B).

**Fig. 4:**
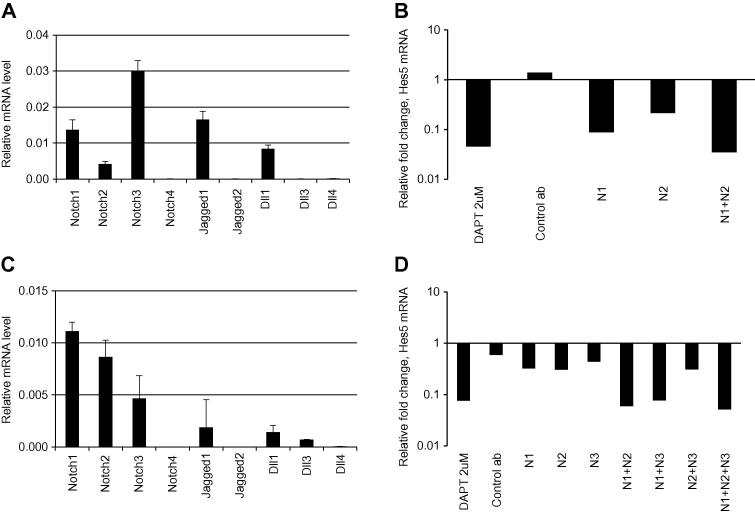
Antibody-mediated inhibition of endogenous mouse and human Notch receptor signalling in neural stem cells. qRT-PCR was used to analyse the relative mRNA levels of Notch pathway genes in neural stem cell systems of both mouse and human origin. (A) Mouse NS cells express several Notch receptors and ligands. (B) Anti-Notch antibodies caused down-regulation of Notch dependent Hes5 expression in mouse NS cells relative to control antibody (Control ab). Inhibition of Hes5 with anti-NRR1 (N1) and anti-NRR2 (N2) antibodies are additive and co-incubation reduces Hes5 to the same extent as DAPT. (C) Notch receptors 1–3 and the ligands JAGGED1 and DLL1 and DLL3 are expressed in human NES cells (AF22). (D) Treating human NES cells with blocking antibodies targeting NRR1 (N1), NRR2 (N2) or NRR3 (N3) reduces HES5 expression. Co-incubation of anti-Notch1 with either anti-Notch2 or anti-Notch3 antibodies reduces HES5 expression to the same extent as inhibition with DAPT. Bars show relative mRNA levels as values normalised to GAPDH based on two separate reactions (A and C). Bars in figure B and D show fold change compared to DMSO control experiments.

This study could not directly examine cross-reactivity with Notch4 since attempts to express the NRR of Notch4 were unsuccessful. This included efforts to make an NRR2 construct in which surface exposed amino acids were substituted with Notch 4 specific sequences (unpublished). While it remains to be experimentally verified that the presented antibodies do not interfere with Notch4 signalling, Notch4 is the most distant member of the receptor family. The protein alignment shown in Supplementary Fig. 4 reveals that any region of homology stretching over more than single residues are shared across multiple family members suggesting, that antibodies which fail to cross react, within the NRR regions of Notch 1–3 (such as N1_E6, N2_B6 and N2_B9,), will not cross react with Notch4. It is also unlikely that Notch4 is involved in neural stem cell differentiation (described below) since previous reports suggest that Notch4 distribution is primary restricted to endothelial cells [35]. In addition expression of Notch4 was not found using qRT-PCR in any of the stem cells analysed in this study (Fig. 4) or in HEK293T cells (data not shown).

### 3.3 Antibody-directed inhibition of endogenous Notch signalling during neural stem cell self-renewal and differentiation

To investigate the effect of antibody-mediated inhibition on endogenous Notch signalling we analysed the relative expression of Hes5, an immediate and highly characterised Notch target gene in mouse and human neural stem cells, using quantitative RT-PCR (qRT-PCR). Initial qRT-PCR demonstrated that Notch 1, 2 and 3 and the ligands Jagged1 and Dll1 were all expressed in mouse neural stem (NS) cells [28] (Fig. 4A). Expression of these family members together with expression of the target gene Hes5 (Fig. 4B) suggested that the Notch pathway is active in these cells. Accordingly, mouse NS cells were cultured for 48 h in media containing 10 μg/ml of blocking antibody before examining the relative expression of Hes5 by qRT-PCR (Fig 4B). In cultures treated with the GSI DAPT, significant down-regulation of Hes5 was observed compared to the DMSO negative control. Analysis of the qRT-PCR revealed a partial reduction in Hes5 expression using individual Notch1 and Notch2 blocking antibodies (N1_E6 and N2_B6) while combined blockade of Notch1 and Notch2 down-regulates Hes5 to the same extent as blockade of all 4 Notches using DAPT (Fig. 4B).

Since the antibodies also bind human Notch receptors (Fig. 2) their utility in blockade of Notch receptors in human NES cells was also examined [27]. qRT-PCR of NES cells (Fig. 4C) revealed that mRNAs of NOTCH receptors 1–3 were expressed and that JAGGED1 and DLL1 were the predominant ligands whereas DLL3 is transcribed at a lower level, suggesting active Notch signalling in these cells similar to pervious findings [36]. As expected, antibody blockade of Notch1 or Notch2 reduce expression of HES5 in these cell cultures (Fig. 4D). A hybridoma derived blocking antibody targeting the NRR of human Notch3 has previously been described [25] and so a synthetic gene encoding the variable heavy and light chains of this Notch3 antibody was synthesised and reformatted as an scFv-Fc-fusion. The resulting antibody (N3_mAb) demonstrated the expected blocking capability against human Notch3 in human NES cells and when applied to cell cultures, a reduced relative level of HES5 was observed compared with controls (Fig. 4D). Again inhibition of single receptor members partially reduce the HES5 expression while blocking of multiple Notch receptors achieves a more pronounced reduction of HES5 level. Combined blockade with antibodies targeting either Notch1 and Notch2 or Notch1 and Notch3 reduced HES5 expression to the same extent as inhibition with DAPT. These results demonstrate that blocking antibodies to Notch1 and Notch2 can be used to interfere with endogenous signalling in mouse and human cell systems and suggests a major contribution of Notch1 to the overall Hes5 expression in both mouse NS cells and human NES cells.

### 3.4 Antibody-mediated inhibition of individual Notch receptors influences fate choice in stem cells

**Fig. 5:**
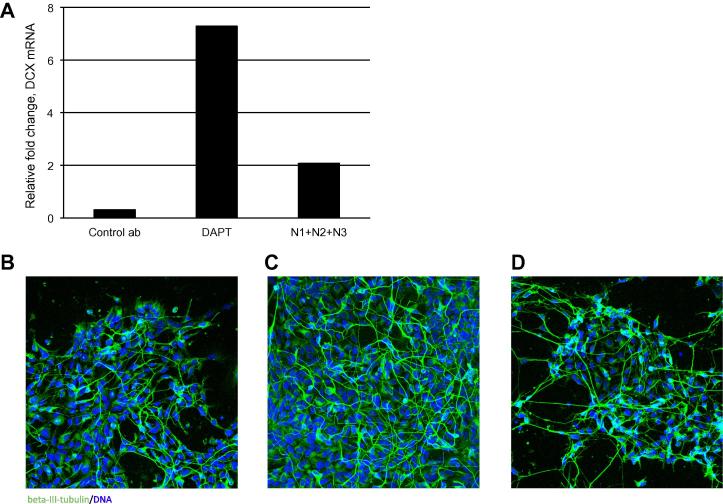
Inhibition of Notch in human NES cells promotes neuronal differentiation. (A) The effect of Notch inhibition on neuronal lineage entry is assessed by qRT-PCR analysis of doublecortin (DCX) expression. Neural stem cells were grown under differentiation conditions for 6 days and RNA was prepared. GAPDH was used for normalisation and values are an average of 2 technical replicates. Data shows representative values of fold-change relative to the DMSO control from one of several independent experiments. Notch specific inhibition with antibodies targeting Notch 1, 2 and 3 generated a 2-fold increase while addition of control antibody had minor effect. Inhibition with the γ-secretase inhibitor DAPT led to a 7-fold increased in DCX expression. (B–D) NES cells were differentiated for 7 days treated with DMSO (B), antibody blockade of Notch1–3 (C) and DAPT (D) for 7 days. Cells were then stained with the post-mitotic neuronal marker beta-III-tubulin (Tuj1) which showed that inhibition of Notch increased the amount of Tuj1 + cells. Experiments were done in duplicates and the proportion of Tuj1 positive cells manually quantified.

We have shown that Notch blocking antibodies inhibit the Notch target gene HES5 and that combining antibodies against multiple Notch receptors increase the inhibitory effect. Previous studies have demonstrated that blocking of Notch signalling in neural stem cells by GSIs causes exit from the stem cell state and induction of differentiation [37]. To determine which individual Notch receptors were responsible for controlling this transition, human NES cells were cultured in the presence of different anti-Notch antibodies. Human NES cells can be stably propagated without loss of stem cell characteristics [27] with differentiation initiated upon withdrawal of EGF and FGF2. Under these differentiation conditions the relative expression of the early neuronal differentiation marker doublecortin (DCX) [38] was analysed using qRT-PCR (Fig 5A). At day 6 of differentiation a 7-fold up-regulation of DCX in cells treated with DAPT was observed. Up-regulation of DCX (2-fold) was measured in cells treated with a combination of blocking antibodies against Notch 1–3. Individual blocking of Notch receptors or combinations of two antibodies have little or no effect on DCX expression.

Expression of beta-III tubulin (Tuj1), a recognised marker for newly committed neurons, was also used to assess the influence of Notch blocking on differentiation outcome in NES cell cultures. After 7 days in differentiating conditions, cell cultures were stained for expression of Tuj1 and positive cells counted (Fig. 5B–D). The proportion of Tuj1 positive cells almost doubled, from 10% in DMSO-treated control cultures (Fig. 5B), when treating with antibodies blocking Notch1, 2 and 3 (Fig. 5C). Although this is not as pronounced as found in cultures treated with DAPT (Fig. 5D), where 25% of cells are Tuj1 positive, it demonstrates that Notch-specific inhibition with blocking antibodies promotes entry into the neuronal lineage. By applying antibodies blocking Notch 1–3 in differentiating human NES cells we see a clear difference in regulation of downstream genes compared to GSI treatment. Thus even though HES5 is reduced to the same extent using DAPT or anti-Notch antibodies, the downstream effect on neural stem cell differentiation is not as pronounced using antibodies. Although further studies are required, this is potentially a reflection of specific Notch inhibition achieved with blocking antibodies in contrast to a general inhibition of all γ-secretase dependent pathways with DAPT.

## 4 Discussion

Many studies of the consequences of Notch signalling fail to distinguish the role of different Notch paralogues although there are clearly differences in expression and function. For example studies involving over-expression of constitutively active ICDs of Notch provides general information on Notch signalling but, given the promiscuity of action of ICDs from different Notch genes, they do not necessarily provide information on the role of the individual receptors. In addition, promiscuity between ligands and receptors limits the use of ligands to probe the role of individual Notch receptors in cell biology studies. Finally commonly used GSIs not only fail to distinguish between different Notch family members, they also influence additional signalling pathways (see below). Thus the existing tools for dissecting the roles of the individual Notch receptors are blunt. Antibodies, on the other hand, have the potential to act as highly specific Notch blocking agents.

Specific blocking antibodies were generated from an antibody phage-display library, by selection on mouse Notch1 and 2. Initial attempts to generate blocking antibodies focussed on the Notch1 LBD centred around EGF-like repeat 12. An antibody which blocked the interaction of Dll4 and Notch1 in an in vitro binding assay was generated, however the potency was relatively modest. These antibodies also failed to block signalling in cell based co-culture assays. In the co-culture assay signalling arises from interaction of receptor and ligand expressed at high levels on adjacent cells and is therefore a consequence of a multivalent interaction. This is, therefore, a more demanding assay than the in vitro ligand binding assay and may explain the difference in results. Blocking could potentially be achieved by using higher concentrations of antibody or by improving the affinity [39].

During the first step of ligand dependent Notch signalling the NRR is converted from a closed inactive state to a more relaxed, activated state induced by ligand binding to the receptor [16,17]. Recent reports have demonstrated that by targeting the NRRs of human Notch receptors it is possible to generate antibodies with antagonistic properties, [25,32,33]. Structural studies suggested that these function by stabilizing the closed receptor conformation and protecting the S2 site from cleavage upon ligand dependent activation. In the present study, antibodies were selected to the NRR region of murine Notch1 and Notch2 by phage display. A cell signalling assay was established where HEK293 cells, expressing either Notch1, 2 or 3, were co-cultured with ligand expressing cells. Ligand/receptor interaction between cells leads to receptor activation that induces expression of a luciferase reporter gene. Using this system we identified blocking antibodies and demonstrated that antibody mediated inhibition was specific and potent with IC_50_ values in the low nM range. Although the anti-Notch antibodies were selected for binding to murine Notch1 and Notch2 we could demonstrate their utility for specific blocking of human Notch1 and 2.

Addition of blocking antibodies to either mouse or human neural stem cells reduced expression of the Notch-dependent gene Hes5. The effect of antibody-mediated inhibition on distinct receptors were additive and simultaneous inhibition of several receptors reduced Hes5 to the same extent as inhibition of γ-secretase. Our work shows that addition of anti-Notch antibodies to cultures of differentiating neural stem cells increased neural differentiation as judged by the upregulation of both beta-III tubulin and doublecortin, supporting a pro-neural effect of blocking Notch signalling. The GSI inhibitor DAPT, also increased expression of doublecortin but to a greater extent. A significant disadvantage of GSIs is that they inhibit signalling through all 4 Notch receptors as well as other γ-secretase dependent pathways. To date, over 60 different proteins have been identified as targets for γ-secretase cleavage [14,40]. In fact several other γ-secretase substrates are important regulators in the neural system, (including amyloid precursor protein (APP) [41], low-density lipoprotein receptor-related protein (LRP)-2 [42], E-cadherin [43], ApoER2 and ErbB-4 [44]). The expanding list of γ-secretase substrates point to the difficulty of using GSIs for investigation of specific cell signalling pathways including Notch. Specific antibody-mediated modulation of individual family members offers strategies to dissect these events in more detail.

Specific inhibition of Notch signalling also presents a promising approach for treatment of diseases such as cancer. GSIs have been tested in various mouse models where they were associated with a reduction in cancer progression and prolonged survival was demonstrated [45]. GSIs, however, also resulted in dose-limiting side effects in vivo. For example pan-specific Notch inhibition decreases proliferation in the intestinal crypts and causes goblet cell metaplasia [46]. The ability to use antibodies to block individual family members has the potential to reduce such side-effects in vivo as previously demonstrated [33].

Antibodies provide a potential route towards greater specificity in controlling signalling of Notch and other signalling systems. In contrast to the widely used chemical inhibitors, our antibodies permit a more precise investigation of the role of individual family members in normal and pathological development/differentiation in both human and mouse systems. While gene knock out approaches provide a means for investigating gene function the availability of blocking antibodies permit reversible and dose dependent studies of Notch function in various model systems without the need for genetic manipulation. The ability to block specific Notch receptors will provide greater understanding of the role of individual family member in differentiation and will permit a greater degree of control of differentiation for both therapeutic and research applications.

## Appendix A Supplementary data

Supplementary Fig. 1 Identification of antibodies targeting NRR1 and NRR2. Individual antibody clones arising from selections on NRR1 (A) or NRR2 (B) were produced and screened in ELISA for target binding. Binding was detected using an anti-FLAG antibody labelled with Europium. TRF signal was measured and antibody clones ranked according to signal intensity. For each target a total of 470 antibody clones were screened (1 out of 5 ELISA plates is shown) and the 96 top-ranked clones (black bars) were chosen for further characterization. TRF intensity is shown as fold change to a negative control (Neg. Ctrl.).

Supplementary Fig. 2 Bivalent antibodies to Notch1 and 2 blocks receptor activation in cell-based Notch signalling assays.Luciferase reporter gene activity reveals receptor activation in Notch expressing cells when these are co-cultured with cells expressing the ligand Jagged1 (Jag+). (A): The 6 blocking anti-NRR1 antibodies have maintained blocking capability of Notch1 in HEK293 cells after sub-cloning to a bivalent scFv-Fc format and expression in mammalian cells. A control antibody (Ctl. Ab.) does not influence receptor activation. Values are an average of 2 replicates. (B): Co-culturing between HEK-Notch2 and HEK-Jag1 cells confirms that the 4 anti-NRR2 antibodies have maintained functionality when produced as bivalent Fc-fusions. DAPT was used as control of Notch inhibition. Luciferase activity in unblocked cultures (Jag+) was used to normalize data in both A and B.

Supplementary Fig. 3 Receptor-specific inhibition of Notch1 and 2 by addition of blocking antibodies to cells cultured on immobilised ligand. Blocking antibodies specifically also inhibit signalling through Notch1 and 2 when immobilised Jagged1 is used to activate the receptors. Cells expressing Notch receptors 1-3 were cultured on immobilised ligand Jagged1 (Jag1-Fc) and receptor activation confirmed by expression of the reporter gene luciferase. (A): Notch1 signalling is efficiently inhibited by the anti-NRR1 antibody N1_E6 but not by antibodies targeting Notch2. (B): Signalling in HEK-Notch2 cells is blocked with anti-NRR2 antibodies N2_B6 and N2_B9 but not with N1_E6. (C): Antibodies directed to Notch1 (N1_E6) or Notch2 (N2_B6 and N2_B9) do not interfere with signalling in cells expressing Notch3. A control antibody targeting the unrelated protein Shc1 has no effect on Notch signalling and was used to normalize the relative luciferase activity. A recombinant human Fc protein (Fc) was used as negative control and illustrates the basal luciferase activity in this assay. All values represent an average of 2 or more experiments.

Supplementary Fig. 4 Sequence alignment of Notch regulatory regions (NRRs) from human and mouse Notch 1-4. (A) Sequence alignment of the NRRs of human (hNRR) and mouse (mNRR) Notch receptors 1-4 reveals the high homology between receptors of both species. (B) The percentage of identical amino acid residues between individual NRRs of human and mouse further highlights the conservation between the 2 species (filled boxes) and that the NRR of Notch4 is the most distant receptor in both species. The degree of conservation is represented by the shading: low/non-similar (white background), conservative (white on grey background), identical (black on grey background).

Supplementary Fig. 5 Verification of Notch expression in reporter cells by flow cytometry. (A) HEK-Notch1 cells are positively stained with Notch 1 antibody N1_E6. The antibodies N2_B9 and N3_E10 which are specific for Notch2 and 3 respectively do not stain these cells. (B) Positive staining with the Notch 2 antibody N2_B9 verifies expression of Notch2 in HEK-Notch2 cells. (C) HEK-Notch3 cells are positively stained for Notch3 with the antibody N3_E10 but not for Notch1 or 2.

Supplementary Fig. 6 Sequences of blocking anti-Notch antibodies. (A) Amino acid sequence of the variable heavy (VH) and variable light (VL) sequences of Notch blocking antibodies N1_E6, N2_B6 and N2_B9 are shown. Positions for complementarity determining regions (CDRs) and framework regions (FW) are indicated. In the comparison between N2_B6 and N2_B9, a dash indicates homology in the 2 sequences. (B) DNA sequence of the above clones in the form of single chain Fvs (scFvs) where VH and VL regions are joined by DNA encoding a flexible linker with the amino acid sequence LEGGGGSGGGGSGGGAS.

Supplementary Table 1

Supplementary Table 2 Primer sequences and probes for qRT-PCR.
