# Supplementary figures and images for: Generation of anti-Notch antibodies and their application in blocking Notch signalling in neural stem cells
Source: Methods. 2012 Sep;58(1):69–78. doi: 10.1016/j.ymeth.2012.07.008 (PMC3502869; doi:10.1016/j.ymeth.2012.07.008)

Supplementary figure 1

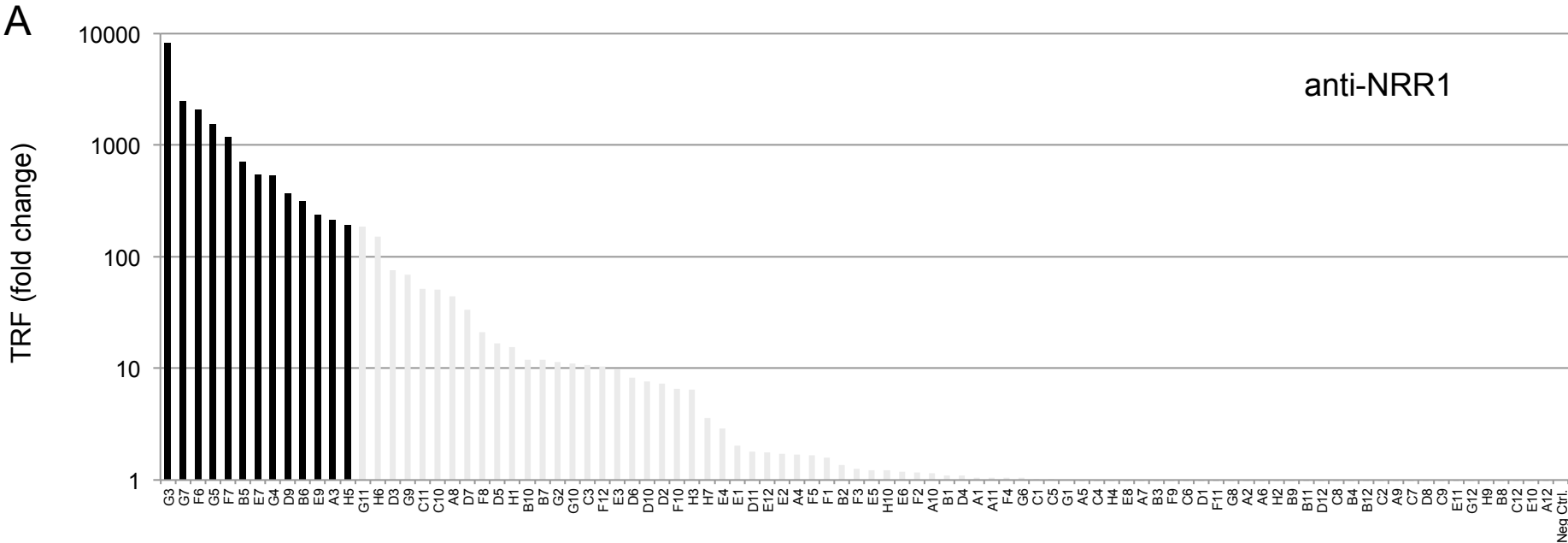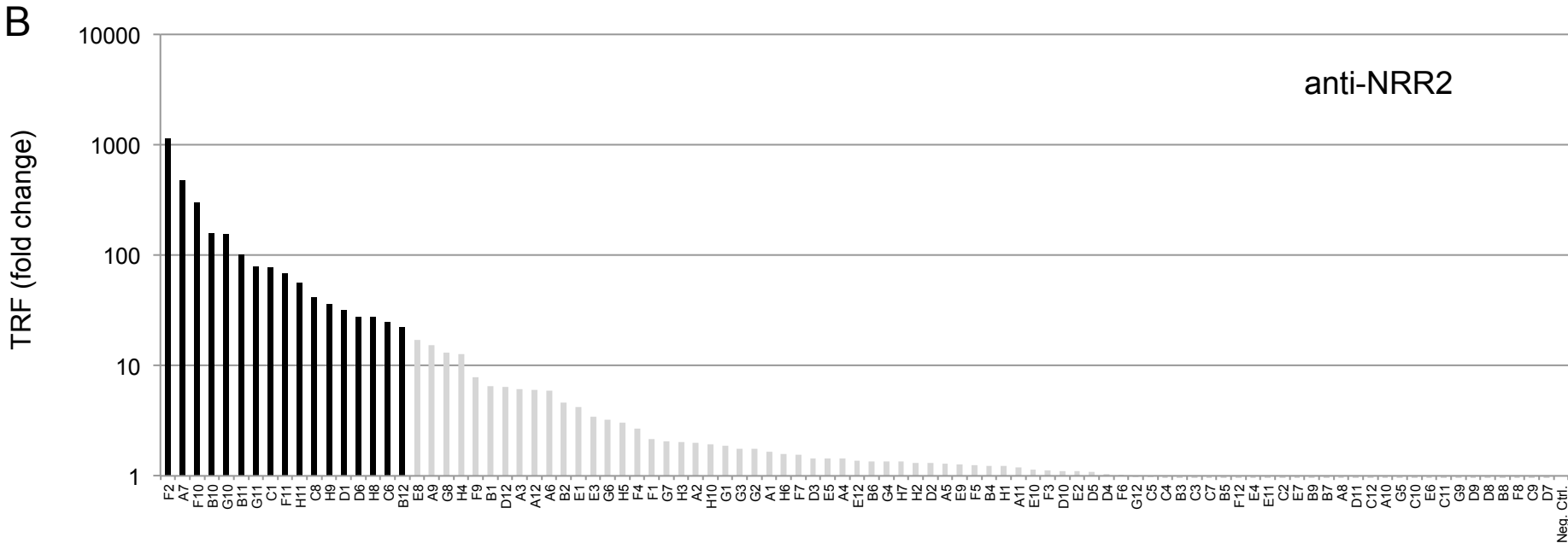

Supplement: Supplementary Fig. 1 — Identification of antibodies targeting NRR1 and NRR2. Individual antibody clones arising from selections on NRR1 (A) or NRR2 (B) were produced and screened in ELISA for target binding. Binding was detected using an anti-FLAG antibody labelled with Europium. TRF signal was measured and antibody clones ranked according to signal intensity. For each target a total of 470 antibody clones were screened (1 out of 5 ELISA plates is shown) and the 96 top-ranked clones (black bars) were chosen for further characterization. TRF intensity is shown as fold change to a negative control (Neg. Ctrl.). [file mmc1.pdf]

## Supplementary figure 2

A

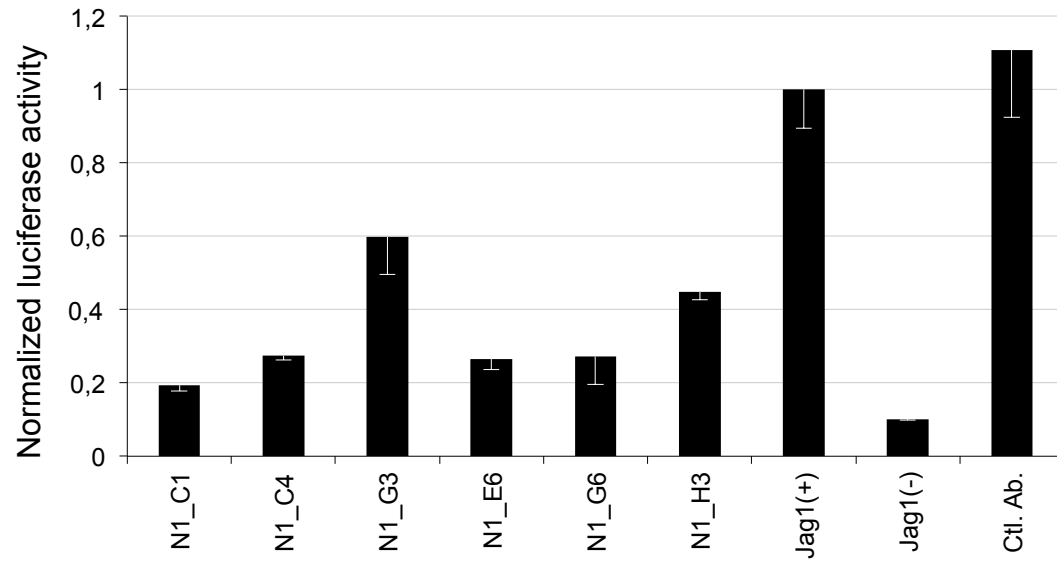

B

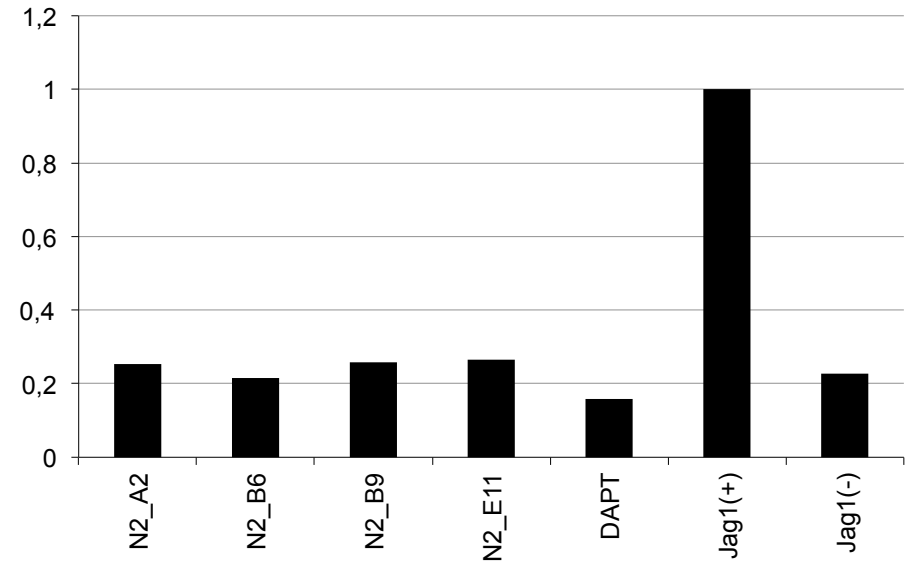

Supplement: Supplementary Fig. 2 — Bivalent antibodies to Notch1 and 2 blocks receptor activation in cell-based Notch signalling assays.Luciferase reporter gene activity reveals receptor activation in Notch expressing cells when these are co-cultured with cells expressing the ligand Jagged1 (Jag+). (A): The 6 blocking anti-NRR1 antibodies have maintained blocking capability of Notch1 in HEK293 cells after sub-cloning to a bivalent scFv-Fc format and expression in mammalian cells. A control antibody (Ctl. Ab.) does not influence receptor activation. Values are an average of 2 replicates. (B): Co-culturing between HEK-Notch2 and HEK-Jag1 cells confirms that the 4 anti-NRR2 antibodies have maintained functionality when produced as bivalent Fc-fusions. DAPT was used as control of Notch inhibition. Luciferase activity in unblocked cultures (Jag+) was used to normalize data in both A and B. [file mmc2.pdf]

# Supplementary figure 3

A

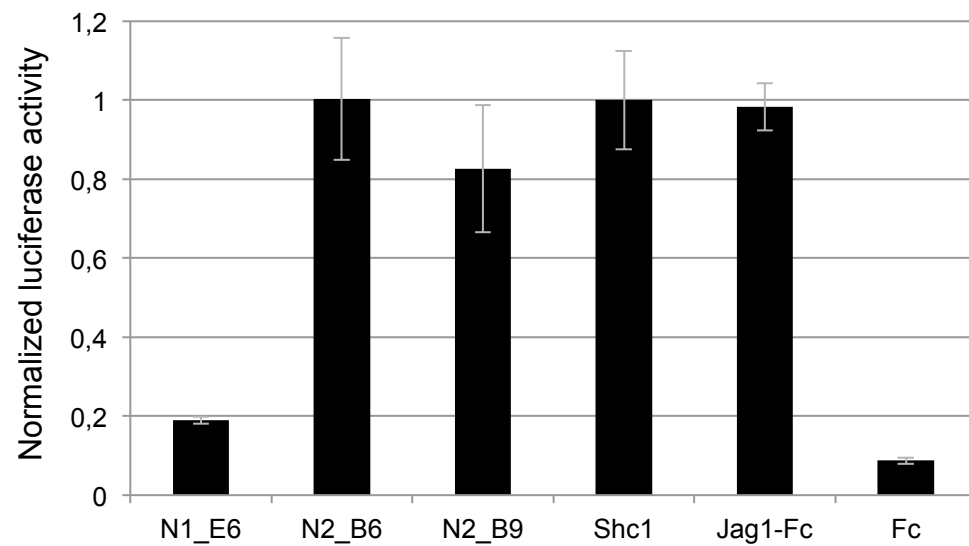

B

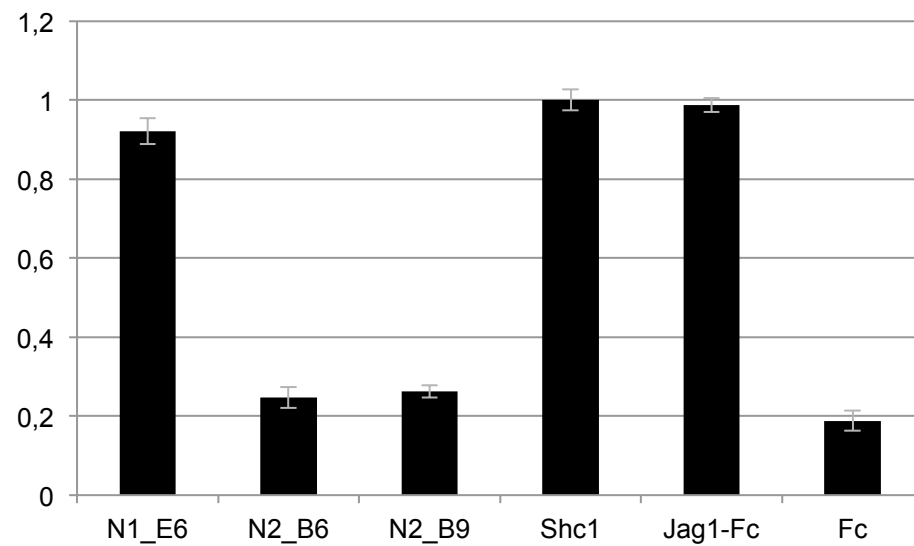

C

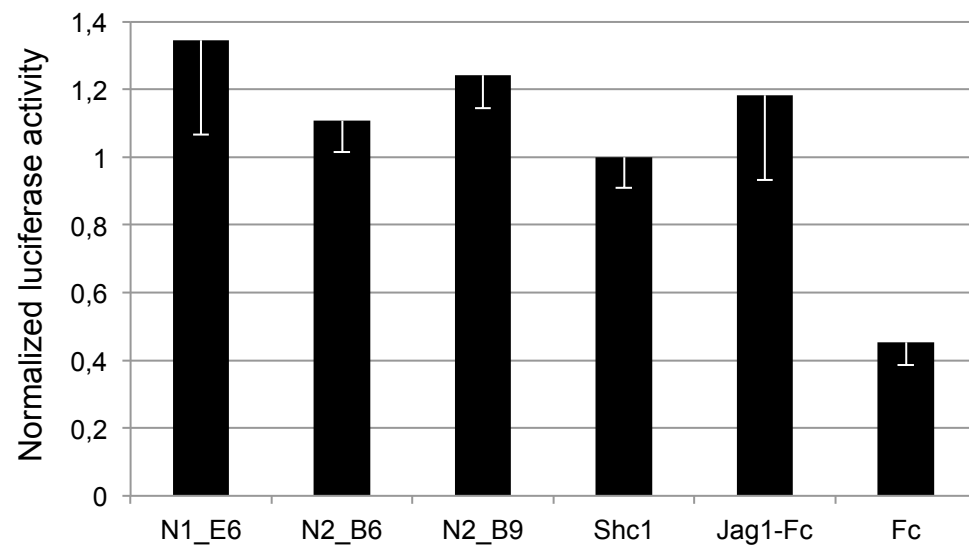

Supplement: Supplementary Fig. 3 — Receptor-specific inhibition of Notch1 and 2 by addition of blocking antibodies to cells cultured on immobilised ligand. Blocking antibodies specifically also inhibit signalling through Notch1 and 2 when immobilised Jagged1 is used to activate the receptors. Cells expressing Notch receptors 1-3 were cultured on immobilised ligand Jagged1 (Jag1-Fc) and receptor activation confirmed by expression of the reporter gene luciferase. (A): Notch1 signalling is efficiently inhibited by the anti-NRR1 antibody N1_E6 but not by antibodies targeting Notch2. (B): Signalling in HEK-Notch2 cells is blocked with anti-NRR2 antibodies N2_B6 and N2_B9 but not with N1_E6. (C): Antibodies directed to Notch1 (N1_E6) or Notch2 (N2_B6 and N2_B9) do not interfere with signalling in cells expressing Notch3. A control antibody targeting the unrelated protein Shc1 has no effect on Notch signalling and was used to normalize the relative luciferase activity. A recombinant human Fc protein (Fc) was used as negative control and illustrates the basal luciferase activity in this assay. All values represent an average of 2 or more experiments. [file mmc3.pdf]

Supplementary figure 5

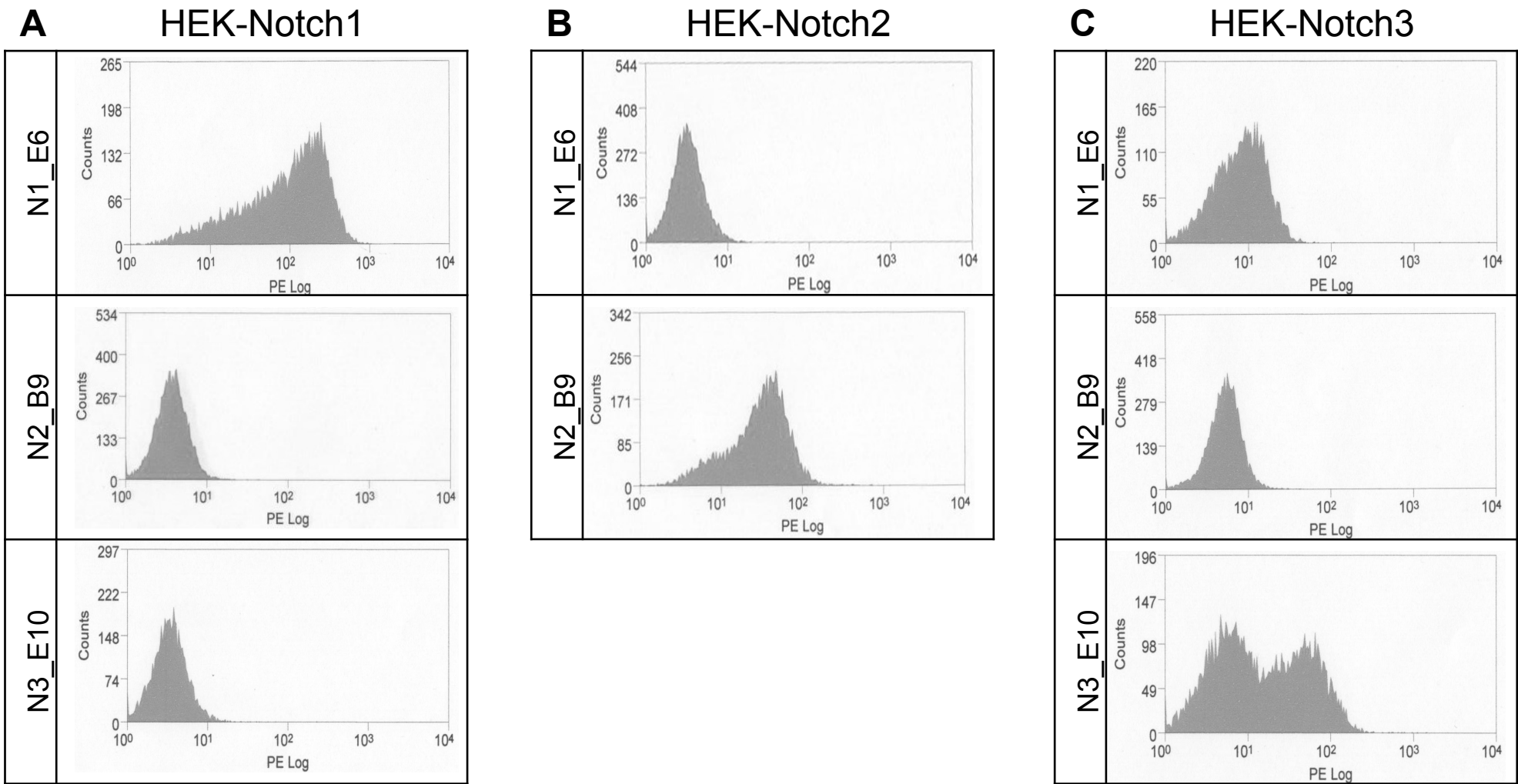

Supplement: Supplementary Fig. 5 — Verification of Notch expression in reporter cells by flow cytometry. (A) HEK-Notch1 cells are positively stained with Notch 1 antibody N1_E6. The antibodies N2_B9 and N3_E10 which are specific for Notch2 and 3 respectively do not stain these cells. (B) Positive staining with the Notch 2 antibody N2_B9 verifies expression of Notch2 in HEK-Notch2 cells. (C) HEK-Notch3 cells are positively stained for Notch3 with the antibody N3_E10 but not for Notch1 or 2. [file mmc5.pdf]
