# Supplementary material for: Generation of anti-Notch antibodies and their application in blocking Notch signalling in neural stem cells
Source: Methods. 2012 Sep;58(1):69–78. doi: 10.1016/j.ymeth.2012.07.008 (PMC3502869; doi:10.1016/j.ymeth.2012.07.008)
Supplement: Supplementary Fig. 4 — Sequence alignment of Notch regulatory regions (NRRs) from human and mouse Notch 1-4. (A) Sequence alignment of the NRRs of human (hNRR) and mouse (mNRR) Notch receptors 1-4 reveals the high homology between receptors of both species. (B) The percentage of identical amino acid residues between individual NRRs of human and mouse further highlights the conservation between the 2 species (filled boxes) and that the NRR of Notch4 is the most distant receptor in both species. The degree of conservation is represented by the shading: low/non-similar (white background), conservative (white on grey background), identical (black on grey background). [file mmc4.pdf]

Supplementary figure 4

A

```
hNRR1   (1) --DYSFGGGAGRDIPPPLIEEACELP---ECQEDAGNKVCSLQCNNHACGWDGGDCSLNFNDPWKNCTQSLQCWKYFSDGHCDSQCNSAGCLFDGFDCQ-
mNRR1   (1) --DYSFTGGAGRDIPPPQIEEACELP---ECQVDAGNKVCNLQCNNHACGWDGGDCSLNFNDPWKNCTQSLQCWKYFSDGHCDSQCNSAGCLFDGFDCQ-
hNRR2   (1) ------------TAPPSTPPATCLSQ---YCADKARDGVCDEACNSHACQWDGGDCSLTMENPWANCSSPLPCWDYINN-QCDELCNTVECLFDNFECQ-
mNRR2   (1) PPSFGGSHCELYTAPTSTPPATCQSQ---YCADKARDGICDEACNSHACQWDGGDCSLTMEDPWANCTSTLRCWEYINN-QCDEQCNTAECLFDNFECQ-
hNRR3   (1) ------RCEAPAAAPEVSEEPRCPRA---ACQAKRGDQRCDRECNSPGCGWDGGDCSLSVGDPWRQC-EALQCWRLFNNSRCDPACSSPACLYDNFDCHA
mNRR3   (1) ------RCETPSAAPEVPEEPRCPRA---ACQAKRGDQNCDRECNTPGCGWDGGDCSLNVDDPWRQC-EALQCWRLFNNSRCDPACSSPACLYDNFDCYS
hNRR4   (1) ----LGGPGFRCSCPHSSPGPRCQKPGAKGCEGRSGDGACDAGCSGPGGNWDGGDCSLGVPDPWKGCPSHSRCWLLFRDGQCHPQCDSEECLFDGYDCE-
mNRR4   (1) -------PGFQCTCPPDSPGPRCQRPGASGCEGRGGDGTCDAGCSGPGGDWDGGDCSLGVPDPWKGCPPHSQCWLLFRDGRCHPQCDSEECLFDGYDCE-

hNRR1  (95) -RAEGQCNPLYDQYCKDHFSDGHCDQGCNSAECEWDGLDCAEHVPERLAAGTLVVVVLMPPEQLRNSSFHFLRELSRVLHTNVVFKRDAHGQQMIFPYYG
mNRR1  (95) -LTEGQCNPLYDQYCKDHFSDGHCDQGCNSAECEWDGLDCAEHVPERLAAGTLVLVVLLPPDQLRNNSFHFLRELSHVLHTNVVFKRDAQGQQMIFPYYG
hNRR2  (84) -GNSKTCK--YDKYCADHFKDNHCDQGCNSEECGWDGLDCAADQPENLAEGTLVIVVLMPPEQLLQDARSFLRALGTLLHTNLRIKRDSQGELMVYPYYG
mNRR2  (96) -RNSKTCK--YDKYCADHFKDNHCDQGCNSEECGWDGLDCASDQPENLAEGTLIIVVLLPPEQLLQDSRSFLRALGTLLHTNLRIKQDSQGALMVYPYFG
hNRR3  (91) GGRERTCNPVYEKYCADHFADGRCDQGCNTEECGWDGLDCASEVPALLARGVLVLTVLLPPEELLRSSADFLQRLSAILRTSLRFRLDAHGQAMVFPYHR
mNRR3  (91) GGRDRTCNPVYEKYCADHFADGRCDQGCNTEECGWDGLDCASEVPALLARGVLVLTVLLPPEELLRSSADFLQRLSAILRTSLRFRLDARGQAMVFPYHR
hNRR4  (96) --TPPACTPAYDQYCHDHFHNGHCEKGCNTAECGWDGGDCRPEDGDPEWGPSLALLVVLSPPALDQQLFALARVLSLTLRVGLWVRKDRDGRDMVYPYPG
mNRR4  (93) --IPLTCIPAYDQYCRDHFHNGHCEKGCNNAECGWDGGDCRPEGEDSEGRPSLALLVVLRPPALDQQLLALARVLSLTLRVGLWVRKDSEGRNMVFPYPG

hNRR1 (194) REEELRKHPIKRAAEGWAAPDALLGQVKASLLPGGSEGGRRRRELDPMDVRGSIVYLEIDNRQCVQA--SSQCFQSATDVAAFLGALASLGSLNIPYKIE
mNRR1 (194) HEEELRKHPIKRSTVGWAT-------S--SLLPGTS-GGRQRRELDPMDIRGSIVYLEIDNRQCVQS--SSQCFQSATDVAAFLGALASLGSLNIPYKIE
hNRR2 (181) E---------KSAAMKKQR------------MTRRSLPGEQEQE-----VAGSKVFLEIDNRQCVQD--SDHCFKNTDAAAALLASHAIQGTLSYPLVSV
mNRR2 (193) E---------KSAAMKKQK------------MTRRSLPEEQEQEQE---VIGSKIFLEIDNRQCVQD--SDQCFKNTDAAAALLASHAIQGTLSYPLVSV
hNRR3 (191) P------------------------------SPGSE--PRARRELAP-EVIGSVVMLEIDNRLCLQSPENDHCFPDAQSAADYLGALSAVERLDFPYPLR
mNRR3 (191) P------------------------------SPGSE--SRVRRELGP-EVIGSVVMLEIDNRLCLQSAENDHCFPDAQSAADYLGALSAVERLDFPYPLR
hNRR4 (194) ARAEEKLGGTRDPTYQER------------AAPQTQPLGKETDSLS----AGFVVVMGVDLSRCGPDHPASRCPWDPGLLLRFLAAMAAVGALEPLLPGP
mNRR4 (191) TRAKEELSGARDSSSWER------------QAPPTQPLGKETESLG----AGFVVVMGVDLSRCGPEHPASRCPWDSGLLLRFLAAMAAVGALEPLLPGP

hNRR1 (292) AVQSETVEPPPPAQ-
mNRR1 (282) AVKSEPVEPPLP---
hNRR2 (253) VSESLTPERTQ----
mNRR2 (267) FSELESPRNAQ----
hNRR3 (258) DVRGEPLEPPEPS--
mNRR3 (258) DVRGEPLEAPEQSV-
hNRR4 (278) LLAVHPHAGTAPP--
mNRR4 (275) LLAAHPQAGTRPSAN
```

B

	mNRR2	mNRR3	mNRR4	hNRR1	hNRR2	hNRR3	hNRR4
mNRR1	46	48	33	90	44	45	33
mNRR2		44	31	45	88	44	32
mNRR3			38	43	44	94	35
mNRR4				33	30	35	83
