# Supplementary material for: Generation of anti-Notch antibodies and their application in blocking Notch signalling in neural stem cells
Source: Methods. 2012 Sep;58(1):69–78. doi: 10.1016/j.ymeth.2012.07.008 (PMC3502869; doi:10.1016/j.ymeth.2012.07.008)
Supplement: Supplementary Fig. 6 — Sequences of blocking anti-Notch antibodies. (A) Amino acid sequence of the variable heavy (VH) and variable light (VL) sequences of Notch blocking antibodies N1_E6, N2_B6 and N2_B9 are shown. Positions for complementarity determining regions (CDRs) and framework regions (FW) are indicated. In the comparison between N2_B6 and N2_B9, a dash indicates homology in the 2 sequences. (B) DNA sequence of the above clones in the form of single chain Fvs (scFvs) where VH and VL regions are joined by DNA encoding a flexible linker with the amino acid sequence LEGGGGSGGGGSGGGAS. [file mmc6.pdf]

A

Heavy chain variable domain (VH)

	FW1	CDR1	FW2	CDR2	FW3	CDR3	FW4
N1_E6	QVQLQQSGPGLVKPSQTLSLTCAISGDSVS	SNSAAWN	WIRQSPSRGLEWLG	RTYYRSKWYNDYAVSVKS	RITINPDTSKNQFSLQLNSVTPEDTAVYYCAR	GGENWGFGFDY	WGQGTLVTVSS
N2_B6	QVQLVQSGAEVKKPGASVKVSCKASGYTFT	SYAMH	WVRQAPGQRLEWMG	WMNAGNGNTKYSQKFQG	RVTITRDTSASTAYMELSSLRSEDTAVYYCAR	DRVPTIPAYRIDY	WGQGTLVTVSS
N2_B9	------------------------------	-----	--------------	-I---------------	--------------------------------	GPRSYGAGGMDV	-----------

Light chain variable domain (VL)

	FW1	CDR1	FW2	CDR2	FW3	CDR3	FW4
N1_E6	QSVLTQPPSASGPPGQRVTISC	SGSRSNIGAYTVN	WYQHLPGTAPKVIIH	SNKQRPS	GVPDRFSGSKSGTSASLAITGLQAEDEADYYC	QSYDSRLRGWV	FGGGTKLTVLG
N2_B6	DIQMTQSPSSVSASVGDRVTITC	RASQGISSWLA	WYQQKPGKAPRLLIY	AASSLQS	GVPSRFSGSGSGTDFTLTISSLQPEDFATYYC	QQANSFPLT	FGGGTKLEIKR
N2_B9	-----------------------	-----------	----------KF---	-------	--------------------------------	---------	------V----

B

```
>N1_E6_scFv
CAGGTACAGCTGCAGCAGTCAGGTCCAGGACTGGTGAAGCCCTCGCAGACCCTCTCACTCACCTGTGCCATCTCCGGGGACAGTGTCTCTAGCAACAGTGCTGCTTGGAACTGGATCAGGCAGTCCCCATCGAGAGGCCTTGAGT
GGCTGGGAAGGACATACTACAGGTCCAAGTGGTATAATGATTATGCAGTATCTGTGAAAAGTCGAATAACCATCAACCCAGACACATCCAAGAACCAGTTCTCCCTGCAGCTGAACTCTGTGACTCCCGAGGACACGGCTGTGTA
TTACTGTGCAAGGGGGGGGGAGAACTGGGGATTCGGGTTTGACTACTGGGGCCAGGGAACCCTGGTCACCGTCTCCTCACTCGAGGGTGGAGGCGGTTCAGGCGGAGGTGGCTCTGGCGGTGGCGCTAGCCAGTCTGTGCTGACT
CAGCCACCCTCAGCGTCTGGGCCCCCGGGCAGAGGGTCACCATCTCGTGTTCTGGAAGCAGGTCCAACATCGGGGCCTATACTGTAAACTGGTACCAGCACCTCCCAGGGACGGCCCCCAAAGTCATCATCCATAGTAATAAGC
AGCGGCCCTCAGGGGTCCCTGACCGATTCTCTGGCTCCAAGTCTGGCACCTCAGCCTCCCTGGCCATCACTGGGCTCCAGGCTGAAGATGAGGCTGATTATTACTGCCAGTCGTATGACAGCAGGCTGCGCGGCTGGGTCTTCGG
CGGAGGGACCAAGCTGACCGTCCTAGGT
```

```
>N2_B6_scFv
CAGGTGCAGCTGGTGCAGTCTGGAGCTGAGGTGAAGAAGCCTGGGGCCTCAGTGAAGGTTTCCTGCAAGGCTTCTGGATACACCTTCACTAGCTATGCTATGCATTGGGTGCGCCAGGCCCCCGGACAAAGGCTTGAGTGGATGG
GATGGATGAACGCTGGCAATGGTAACACAAAATATTCACAGAAGTTCCAGGGCAGAGTCACCATTACCAGGGACACATCCGCGAGCACAGCCTACATGGAGCTGAGCAGCCTGAGATCTGAAGACACGGCTGTGTATTACTGTGC
GAGGGATCGCGTGCCTACGATCCCCGCCTATAGAATTGACTACTGGGGCCAGGGAACCCTGGTCACCGTCTCCTCACTCGAGGGTGGAGGCGGTTCAGGCGGAGGTGGCTCTGGCGGTGGCGCTAGCGACATCCAGATGACCCAG
TCTCCATCTTCCGTGTCTGCATCTGTAGGAGACAGAGTCACCATCACTTGTCGGGCGAGTCAGGGTATTAGCAGCTGGTTAGCCTGGTATCAGCAGAAACCAGGGAAAGCCCCTAGGCTCCTGATCTATGCTGCATCCAGTTTGC
AAAGTGGGGTCCCATCAAGGTTCAGCGGCAGTGGATCTGGGACAGATTTCACTCTCACCATCAGCAGCCTGCAGCCTGAAGATTTTGCAACTTACTATTGTCAACAGGCTAACAGTTTCCCGCTCACTTTCGGCGGAGGGACCAA
GCTGGAGATCAAACGT
```

```
>N2_B9_scFv
CAGGTCCAGCTTGTGCAGTCTGGGGCTGAGGTGAAGAAGCCTGGGGCCTCAGTGAAGGTTTCCTGCAAGGCTTCTGGATACACCTTCACTAGCTATGCTATGCATTGGGTGCGCCAGGCCCCCGGACAAAGGCTTGAGTGGATGG
GATGGATCAACGCTGGCAATGGTAACACAAAATATTCACAGAAGTTCCAGGGCAGAGTCACCATTACCAGGGACACATCCGCGAGCACAGCCTACATGGAGCTGAGCAGCCTGAGATCTGAAGACACGGCTGTGTATTACTGTGC
GAGAGGGCCGAGGAGCTATGGCGCGGGCGGTATGGACGTCTGGGGCCAAGGCACCCTGGTCACCGTCTCTTCACTCGAGGGTGGAGGCGGTTCAGGCGGAGGTGGCTCTGGCGGTGGCGCTAGCGACATCCAGATGACCCAGTCT
CCATCTTCTGTGTCTGCATCTGTAGGAGACAGAGTCACCATCACTTGTCGGGCGAGTCAGGGTATTAGCAGCTGGTTAGCCTGGTATCAGCAGAAACCAGGGAAAGCCCCTAAGTTCCTGATCTATGCTGCATCCAGTTTGCAAA
GTGGGGTCCCATCAAGGTTCAGCGGCAGTGGATCTGGGACAGATTTCACTCTCACTATCAGCAGCCTGCAGCCTGAAGATTTTGCAACTTACTATTGTCAACAGGCTAACAGTTTCCCGCTCACTTTCGGCGGAGGGACCAAGGT
GGAAATCAAACGT
```
