# Supplementary material for: Generation of anti-Notch antibodies and their application in blocking Notch signalling in neural stem cells
Source: Methods. 2012 Sep;58(1):69–78. doi: 10.1016/j.ymeth.2012.07.008 (PMC3502869; doi:10.1016/j.ymeth.2012.07.008)
Supplement: Supplementary Table 1 [file mmc7.pdf]

Supplementary table 1.

Gene name (Accession)	Primer	Sequence (given in 5´ to 3´ orientation)
mouse Notch1 (NM_008714)	F mNRR1[a]	CTTTCTACCGCTGTCTATGCCCC
	R mNRR1[a]	GCCCACAAAGAACAGGAGCAC
	F mNRR1[b]	AAAAAGCAGGCTCTGACTACAGCTTCACAGGTGGC
	R mNRR1[a]	AGAAAGCTGGGTTGGGCAGCGGAGGCTCC
	F mNRR1dS1	pho-GAGCTGGACCCCATGGACATC
	R mNRR1dS1	pho- CCCACCACTGGTACCAGGAAG
mouse Notch2 (NM_010928)	F mNRR2[a]	GTAACCCCTGCCAGCATGGG
	R mNRR2[a]	CCATGATGACCCCCAGCAGG
	F mNRR2[b]	AAAAAGCAGGCTCTCCTCCGTCGTTCGGGGG
	R mNRR2[b]	AGAAAGCTGGGTTCTGGGCGTTTCTTGGACTCTC
human Notch1 (NM_017617)	F hNRR1[a]	ACGGGCTCTTGTGCCACATCC
	R hNRR1[a]	CTTGCGGGACAGCAGCACC
	F hNRR1[b]	AAACTCGAGGACTACAGCTTCGGGGGTG
	R hNRR1[b]	AAATGCGGCCGCGTGCAGCTGCGCCGGC
	F hNRR1dS1	pho-GAGCTGGACCCCATGGACG
	R hNRR1dS1	pho-CCCACCCTCGCTGCCAC
Human Notch2 (NM_024408)	F hNRR2[a]	CAGCTGCCACCCTCAGCG
	R hNRR2[a]	TTTTGCCATGATTACCCCCAGCAG
	F hNRR2[b]	AAACTCGAGACGGCACCCCCCAGCAC
	R hNRR2[a]	AAATGCGGCCGCCTGAGTGCGTTCTGGAGTCAG

Table 1. F = forward primer, R = reverse primer, [a]outer primer, [b]nested primer, pho = phosphorylated
