# Supplementary material for: Generation of anti-Notch antibodies and their application in blocking Notch signalling in neural stem cells
Source: Methods. 2012 Sep;58(1):69–78. doi: 10.1016/j.ymeth.2012.07.008 (PMC3502869; doi:10.1016/j.ymeth.2012.07.008)
Supplement: Supplementary Table 2 — Primer sequences and probes for qRT-PCR. [file mmc8.pdf]

Marker/gene	Forward primer	Reverse primer	UPL probe
hGAPDH	ctctgctcctcctgttcgac	acgaccaaatccgttgactc	60
hHES5	tacctgaagcacagcaaagc	tagtcctggtgcaggctctt	70
hNOTCH1	cgcacaaggtgtcttccag	ccctggcaatgtacttgtgat	85
hNOTCH2	tcggcagactggtgacttc	acaggtgctcccttcaaaac	8
hNOTCH3	gccaagcggctaaaggta	cactgacggcaatccaca	30
hNOTCH4	aggccacagcaggatcac	aggtggggccttcaaaac	43
hJAGGED1	gaatggcaacaaaacttgcat	agccttgtcggcaaatagc	42
hJAGGED2	tgggactgggacaacgatac	atgcgacactcgctcgat	17
hDLL1	cttccccttcggcttcac	gggttttctgttgcgaggt	2
hDLL3	caactgtgagaagagggtgga	ccaggtccaggcagagtc	46
hDLL4	ccctggcaatgtacttgtgat	tggtgggtgcagtagttgag	23
hDCX	catccccaacacctcagaag	ggaggttccgtttgctga	78
mGAPDH Taqman Probe (ABI)	Mm99999915_g1		
mHes5	ccaaggagaaaaaccgactg	tgctctatgctgctgttgatg	22
mNotch1	actatctcggcggcttttc	ggcactcgttgatctcctct	5
mNotch2	tgcctgtttgacaactttgagt	gtggtctgcacagtatttgtcat	6
mNotch3	agctgggtcctgaggtgat	agacagagccggttgtcaat	9
mNotch4	ggacctgcttgcaaccttc	cctcacagagcctcccttc	34
mJagged1	gaggcgtcctctgaaaaaca	acccaagccactgttaagaca	6
mJagged2	gctttgtgatcgagcatcc	cttgcagggctgaaagaca	10
mDll1	ttcaactgtgagaagaagatggat	gccgaggtccacacactt	103
mDll3	tcgtacgtgtgcccttcc	tgctctctccaggtttcaatg	7
mDll4	aggtgccacttcggttacac	gggagagcaaatggctgata	106

Supplementary Table 2. Primer sequences and probes for qRT-PCR.
